# Regional analysis of volumes and reproducibilities of automatic and manual hippocampal segmentations

**DOI:** 10.1371/journal.pone.0166785

**Published:** 2017-02-09

**Authors:** Fabian Bartel, Hugo Vrenken, Fetsje Bijma, Frederik Barkhof, Marcel van Herk, Jan C. de Munck

**Affiliations:** 1 Department of Physics and Medical Technology, VU University Medical Center, Amsterdam, The Netherlands; 2 Department of Radiology, VU University Medical Center, Amsterdam, The Netherlands; 3 Department of Mathematics, VU University Amsterdam, Amsterdam, The Netherlands; 4 Image Analysis Center, VU University Medical Center, Amsterdam, The Netherlands; 5 Department of Radiotherapy Physics, University of Manchester, Manchester, United Kingdom; Mathematical Institute, HUNGARY

## Abstract

**Purpose:**

Precise and reproducible hippocampus outlining is important to quantify hippocampal atrophy caused by neurodegenerative diseases and to spare the hippocampus in whole brain radiation therapy when performing prophylactic cranial irradiation or treating brain metastases. This study aimed to quantify systematic differences between methods by comparing regional volume and outline reproducibility of manual, FSL-FIRST and FreeSurfer hippocampus segmentations.

**Materials and methods:**

This study used a dataset from ADNI (Alzheimer’s Disease Neuroimaging Initiative), including 20 healthy controls, 40 patients with mild cognitive impairment (MCI), and 20 patients with Alzheimer’s disease (AD). For each subject back-to-back (BTB) T1-weighted 3D MPRAGE images were acquired at time-point baseline (BL) and 12 months later (M12). Hippocampi segmentations of all methods were converted into triangulated meshes, regional volumes were extracted and regional Jaccard indices were computed between the hippocampi meshes of paired BTB scans to evaluate reproducibility. Regional volumes and Jaccard indices were modelled as a function of group (G), method (M), hemisphere (H), time-point (T), region (R) and interactions.

**Results:**

For the volume data the model selection procedure yielded the following significant main effects G, M, H, T and R and interaction effects G-R and M-R. The same model was found for the BTB scans. For all methods volumes reduces with the severity of disease.

Significant fixed effects for the regional Jaccard index data were M, R and the interaction M-R. For all methods the middle region was most reproducible, independent of diagnostic group. FSL-FIRST was most and FreeSurfer least reproducible.

**Discussion/Conclusion:**

A novel method to perform detailed analysis of subtle differences in hippocampus segmentation is proposed. The method showed that hippocampal segmentation reproducibility was best for FSL-FIRST and worst for Freesurfer. We also found systematic regional differences in hippocampal segmentation between different methods reinforcing the need of adopting harmonized protocols.

## Introduction

The hippocampus is an important brain structure that plays a crucial role in episodic memory [1]. For instance, longitudinal decline of hippocampal volume is related to memory impairment and clinical dementia [2,3]. In Alzheimer’s disease (AD) and its prodromal phase, mild cognitive impairment (MCI), the hippocampus is affected by amyloid and tau pathology early in the disease course [4,5]. Hippocampal atrophy as measured on T1-weighted volumetric structural magnetic resonance images (MRI) is a sensitive biomarker of AD pathology [6], but can also be a predictive imaging biomarker of MCI [7]. Knowledge of hippocampal shape is also an important aspect in radiotherapy, when prophylactic cranial irradiation (PCI) is used and hippocampal avoidance is executed to limit neurocognitive toxicity [8–12].

Although manual outlining by experts is considered as the gold standard, it requires extensive training and is very labour intensive [13]. Therefore, automatic segmentation tools based on deformable models, single-, multiple- or probabilistic-atlases have been developed over the last decades. V. Dill and colleagues give an excellent overview of semi-automatic and automatic hippocampus segmentation methods [14]. The most commonly used publicly available software tools to the academic community, with active user communities and active support from the developers, are FreeSurfer [Martinos Center for Biomedical Imaging, Harvard-MIT, Boston USA] [15,16] and FSL-FIRST [FMRIB Integrated Registration and Segmentation Tool, University of Oxford, Oxford UK] [17] and therefore we focus on these methods. Previous studies have shown good but not perfect overall agreement for both methods with manual segmentation, given a dice overlap of FreeSurfer and FSL-FIRST segmentation ranging from 74–82% and 79–84% respectively and a good volume correlation of both methods with manual segmentation [16–28]. In a direct comparison, FreeSurfer slightly agreed better with manual segmentation than FSL-FIRST [29–33].

So far, most studies comparing manual and automatic hippocampus segmentations have expressed the performance of hippocampus outline methods in terms of global hippocampal volumes and overlap indices to manual hippocampus segmentation. For instance, Mulder and colleagues compared reproducibility of longitudinal hippocampal volume changes, as determined by manual segmentations, FSL-FIRST and FreeSurfer [33]. However, volumes and volume changes do not contain information about shape and overlap indices only quantify the total amount of agreement of two segmentation methods. It is very likely that some parts of the hippocampal structure are easier to segment than others and therefore to study systematic differences existing global volume and overlap measures need to be extended to regional ones. Following Hackert and colleagues we focus on regional differences along the long axis of hippocampi, computing regional volumes and outline reproducibilities by dividing the hippocampus in three regions, the head, body and tail [34]. Furthermore, different automatic segmentation methods and manual segmentation protocols might be based on different underlying anatomical definitions. A systematic regional comparison can reveal such differences between methods.

There are a few cross-sectional hippocampus studies using FreeSurfer segmentation which reported that sub-regions undergo differential atrophy in AD [35][36]. These findings further motivate our objective to evaluate regional longitudinal changes in hippocampal volume as determined by different segmentation methods.

To our knowledge, there are no papers reporting reproducibility of hippocampal outlines in a dataset similar to clinical trials. In part the absence of such studies derives from the fact that comparing voxel-wise segmentations obtained from different scans is challenging, because of slightly different positions of the head in the voxel space. Considering these small regional differences between different segmentations, we wish to avoid interpolation errors as much as possible. For that purpose, in this study a surface reconstruction of each hippocampus is derived from the scan to which the labelled segmentation was available in its rawest form. Then, after determining the accurate image registration and applying the corresponding transformation parameters between the reconstructed surfaces overlap measures were computed directly on the surfaces, avoiding interpolation errors as much as possible. Since the limiting factor of these computations is accuracy of the image registration we apply the “full circle method” to test the quality of registration procedures [37].

It remains unclear to what extent the hippocampal segmentations themselves are reproducible at the most detailed level. Although accuracy of hippocampal segmentations has been investigated by comparing to manual references [17,20–22,27,29], reproducibility of the segmentations has not been investigated on a large population and different groups. Similar to Mulder and colleagues we investigate hippocampus segmentation for different disease groups in different stages and use different segmentation methods [33]. But different to [33], we compare hippocampal volumes and outline reproducibilities in different regions and hemispheres as determined in baseline and follow-up scans. Because of the many factors and possible combination of factors that may influence the response variables, we propose a novel method, based on Akaike Information Criterion (AIC) [38], to select the most suitable statistical model to explain our findings. We test the robustness of this method by performing the same analysis making use of the back-to-back (BTB) scans.

## Materials and methods

### Dataset and MRI acquisition

Data used in the preparation of this article were obtained from the Alzheimer’s Disease Neuroimaging Initiative (ADNI) database (adni.loni.usc.edu). The ADNI was launched in 2003 as a public-private partnership, led by Principal Investigator Michael W. Weiner, MD. The primary goal of ADNI has been to test whether serial magnetic resonance imaging (MRI), positron emission tomography (PET), other biological markers, and clinical and neuropsychological assessment can be combined to measure the progression of mild cognitive impairment (MCI) and early Alzheimer’s disease (AD).

The dataset used in this study is the same subset of the ADNI dataset that has been used by Mulder and colleagues [33]. MRI data of 80 subjects were selected, of which 20 are control subjects (CTRL), 40 MCI subjects and 20 subjects were diagnosed as AD. MCI subjects were a priori selected based on their cerebrospinal fluid (CSF) profile. For the selection we used the ratio of total tau (t-tau) and Amyloid-β 1 to 42 peptide (Aβ_1–42_) with an AD-positive cut-off value of t-tau/Aβ_1–42_ ≥ 0.39 determined by Shaw and colleagues [39]. 20 MCI subjects with an AD-positive cut-off value (MCI-P; t-tau/Aβ_1–42_ ≥ 0.39) and 20 MCI subjects with an AD-negative cut-off value (MCI-N; t-tau/Aβ_1–42_ < 0.39) were selected from the database. All healthy controls had a t-tau/Aβ_1–42_ < 0.39 and all AD’s a t-tau/Aβ_1–42_ ≥ 0.39.

For all subjects four volumetric MRI scans were acquired, two scans at time-point baseline (BL) and two scans one year later, here referred to as M12. Those two MRI BTB scans at each time-point were acquired in a single session, with the acquisition of the second volumetric MRI starting only a few minutes after completing the first acquisition. We refer to these scans as BL-A, BL-B, M12-A, and M12-B. BL scans of all subjects were made between September 2005 and August 2007.

MRI scans were acquired at different locations with 1.5T scanners from various vendors (Philips, Siemens and GE). For every subject the MRI scanner and protocols were the same for each of the four acquisitions. The images were acquired with a 3D T1 weighted magnetization prepared rapid acquisition gradient echo sequence (MPRAGE). All pixels were square and the slice thickness was 1.2mm. The voxel volume ranged from 1.05mm^3^ to 2.03mm^3^ with a median value of 1.88mm^3^. The MRI scans were visually inspected for their quality and no post-processing other than default scanner corrections were performed. A more detailed description for the MRI acquisition protocol can be found in Jack et al [40].

### Hippocampus segmentation

#### Manual

**Fig 1:**
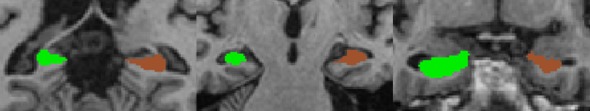
Hippocampus segmentation in reformatted pseudo coronal orientation. Brown colour is the left, green the right hippocampus. Left: posterior slice close to the crux of the fornix. Middle: one of the middle slices of the hippocampus. Right: anterior slice with hippocampus next to the amygdala.

Manual hippocampus segmentations were performed in the Image Analysis Center (IAC, Amsterdam) using their standard operating procedure (SOP) as previously described in [33,41,42]. BL scans were reformatted in a plane perpendicular to the long axis of the left hippocampus, resulting in a pseudo coronal orientation with a slice thickness of 2mm and the original in-plane resolution using sinc interpolation. This procedure was followed independently for all four scans. Rigid body registration was applied to all four (both BL and both M12) reformatted scans to bring them in the same coordinate space for comparison. Three slices of a hippocampus segmentation in pseudo coronal orientation are shown in Fig 1.

Included in the hippocampal formation are the Ammon’s horn, dentate gyrus, alveus and fimbria and the subiculum. To summarize hippocampal boundaries, the most posterior slice is chosen such the total length of the crux of the fornix is seen. The medial boundary of the hippocampus is formed by the CSF in the cisterna ambiens and the transverse fissure. The inferior border is formed by the subiculum and the parahippocampal gyrus. The superior border is defined by the CSF of the temporal horn and the alveus. Laterally, the hippocampus is bordered by CSF from the temporal pol of the lateral ventricle. In anterior direction it forms along the amygdala and stops when an additional amount of CSF appears on the medial side of the hippocampus.

One trained expert technician from the IAC segmented the left and right hippocampus of all subjects using a locally developed software package (Show_Images 3.7.1.0) from the VU University Medical Center (VUmc). The technician was blinded to the diagnosis, but used BL segmentations to segment the follow up M12 scans, as it is part of the workflow of the longitudinal study. However, first and second BTB scans were given in a random order.

#### FSL-FIRST

In [43] and [17] technical details of FSL-FIRST are described. FSL-FIRST is a deformable model based segmentation tool, using shape and appearance models which were constructed from a set of manual segmented subjects provided by the Center for Morphometric Analysis (CMA), Massachusetts General Hospital (MGH), Boston. The manual segmentations were parameterized and described as surface meshes from which a point distribution is modelled. Using observed intensity values from the MR image, FSL-FIRST finds the most probable shape by searching through linear combinations of shape variation modes. FSL-FIRST uses a two-stage affine transformation to a MNI152 standard space of 1mm resolution before performing segmentation. Hippocampus meshes are then converted to labelled voxel region of interests (ROI) after a boundary correction using FAST voxel-wise segmentation software [44]. We used FSL-FIRST v.5.0.4 and the *run_first_all* script command, because FSL-FIRST takes adjacent structures into account. The voxelwise labelled hippocampus segmentation produced by FSL-FIRST are in native MRI scan space.

For one subject the FSL-FIRST segmentation failed because of an internal registration problem. To include this subject, we pre-processed it by extracting the subjects brain using BET before running the FSL-FIRST script. The BET extraction corrected the registration problem and enabled us to include this subject.

#### FreeSurfer

In [16] the technical procedure for subcortical segmentation is described in detail. Briefly, FreeSurfer brings the MRI to a conformed 1mm^3^ 256^3^ space, performs intensity normalization to correct for intensity non-uniformity in the MR image, saves an affine transformation to Talairach space, corrects intensity fluctuations using another normalization and strips the skull leaving only the brain. To apply segmentation labels FreeSurfer transforms the subject’s volume to the FreeSurfer atlas and assigns voxels to subcortical structures using prior probabilistic intensity and tissue class information.

We used the FreeSurfer version 5.3 to perform hippocampus segmentations using the longitudinal processing stream. This requires a prior cross-sectional processing of each MRI. FreeSurfer’s labelled hippocampus segmentations from the cross-sectional and longitudinal stream were converted back to the native MR image space using the procedure provided by FreeSurfer (mri_label2vol).

#### Surface extraction

All volumetric hippocampi labels from each method were converted to triangulated meshes with the marching cube algorithm to avoid interpolation errors introduced by registrations. Those generated hippocampi meshes were used to compute regional volumes and outline reproducilbities. If the segmentations consisted of multiple connected components the surface reconstruction would also consists of multiple surfaces of which the total volume was taken to correspond to the hippocampus.

### Comparison methods

The marching cubes algorithm applied to the segmented images resulted in closed triangulated surfaces. Regional volumes from surfaces were computed by adopting a fine regular grid enclosing two surfaces *A* and *B*, and by testing for each point whether it was inside either of the surfaces. To speed up these computations, KD trees and some other optimizations were used [45]. The Jaccard index of the surface pair (A, B), defined as

$$Jacc(A,B)=\frac{\left|A\cap B\right|}{\left|A\cup B\right|}$$ (1)

was approximated as

$$Jacc(A,B)\approx \frac{N(A\cap B)}{N(A\cup B)}$$ (2)

where *N*(*V*) is the number of grid points inside surface *V*. These grid points were derived from a submillimetre mesh that was fine enough to capture all surface details.

**Fig 2:**
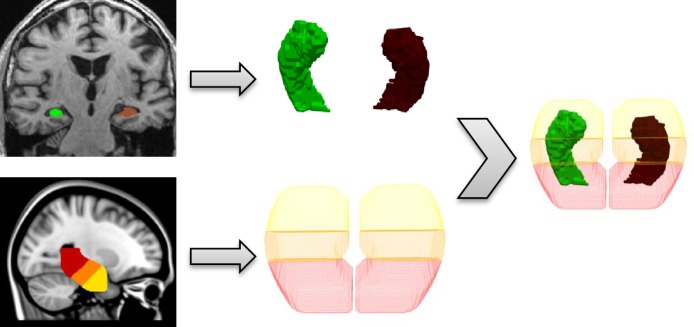
Procedure to make a regional analysis. Top and bottom rows show the conversion from a hippocampus segmentation and the six regional mask to a triangulated mesh respectively. The right part of the figure illustrates the registration procedure to map the six regional hippocampus mask to the left and right hippocampus mesh.

To quantify regional specific reproducibility and systematic differences in shape definition, a regional overlap index was computed as follows:

$${Jacc}_{ROI}=\frac{\left|(A\cap B)\cap ROI\right|}{\left|\left(A\cap ROI)\cup (B\cap ROI\right)\right|}$$ (3)

where ROI represents a region of interest. This equation is an overlap between surfaces *A* and *B*, both constrained to a third region ROI. To compute regional volumes and Jaccard indices in practice, a hippocampus mask was derived from MNI152 standard-space provided by FSL (http://fsl.fmrib.ox.ac.uk/fsl/fslwiki/Atlases, MNI152_T1_1mm_Hipp_mask_dil8.nii). This mask was big enough to cover any hippocampus and was split into three parts for each hemisphere along the long hippocampal axis and converted to triangulated meshes resulting into six mesh regions, hereafter named left and right anterior, middle and posterior. The regions have no specific anatomical definition, but they are similar to Hackert and colleagues’ regional definition and approximate to an anterior region of 35%, middle region of 45% and posterior region of 20% [34]. To register this six regional hippocampus mask in MNI152 space to each subject image space, we performed a similar procedure as FSL-FIRST, i.e. brain extraction, a two-stage affine registration to MNI152, followed by visual inspection. Fig 2. is a flowchart illustrating the hippocampal mesh conversion and the registration procedure of the six regional mask to the hippocampus mesh. All other triangulated hippocampi segmentation meshes (BL-B, M12-A and M12-B) were rigid body registered to scan BL-A with the registration matrices described in *Registrations and registration quality control*.

### Pre-processing

Before regional volumes and reproducibilities could be computed, MRI scans were mapped to each other so that the segmentations were in the same imaging space. Although BTB scans are very similar to the original, there is still the possibility of subject motion in between the BTB scans, and therefore image registration was also applied between these image pairs.

#### Registrations and registration quality control

Rigid body transformations were used to map BL-B to BL-A, M12-B to M12-A and M12-A scan to BL-A scan. Our registrations were all performed using FSL-FLIRT. To check the quality of these registrations, a consistency test was done on the registration parameters using the full circle method introduced by van Herk and colleagues [37]. By registering images in a cyclic fashion and multiplying all transformation matrices the product should result in the identity matrix, when registration errors were absent. Hence, we computed the “full circle” matrix *RM* = ∏*T*_*ij*_, where *T*_*ij*_ is the transformation from image *i* to image *j*. We analysed four full circles which resulted into residual matrices given by:

$${RM}_{Circle1}=T_{BLA-M12B}\times T_{M12B-M12A}\times T_{M12A-BLA}$$ (4)

$${RM}_{Circle2}=T_{BLA-M12A}\times T_{M12A-BLB}\times T_{BLB-BLA}$$

$${RM}_{Circle3}=T_{BLA-M12B}\times T_{M12B-BLB}\times T_{BLB-BLA}$$

$${RM}_{Circle4}=T_{M12B-M12A}\times T_{M12A-BLB}\times T_{BLB-M12B}$$

and determined the residual translation and rotation errors as:

$${Translation}_{Total}=\|TransVector(RM)\|$$ (5)

$${Rotation}_{Total}=\mathrm{acos}\left(\frac{Trace(RM)-1}{2}\right)$$ (6)

In addition, the effect of registration errors was directly quantified by computing the Jaccard index between a hippocampal surface and its transformed version obtained by applying RM:

$$Consistency=Jacc(S_{hip},RM(S_{hip}))$$ (7)

The more consistent all registrations, the closer the matrix RM is to the identity, and the higher this Consistency index. Therefore, we use 1- Consistency to quantify the registration error.

#### Visual quality control

Next to the full circle analysis as an additional quality check, we inspected the results of the outline reproducibility analysis and visually reviewed subjects’ registered scan pairs which had low Jaccard indices to be sure that there were no registration errors.

### Statistical analysis

**Fig 3:**
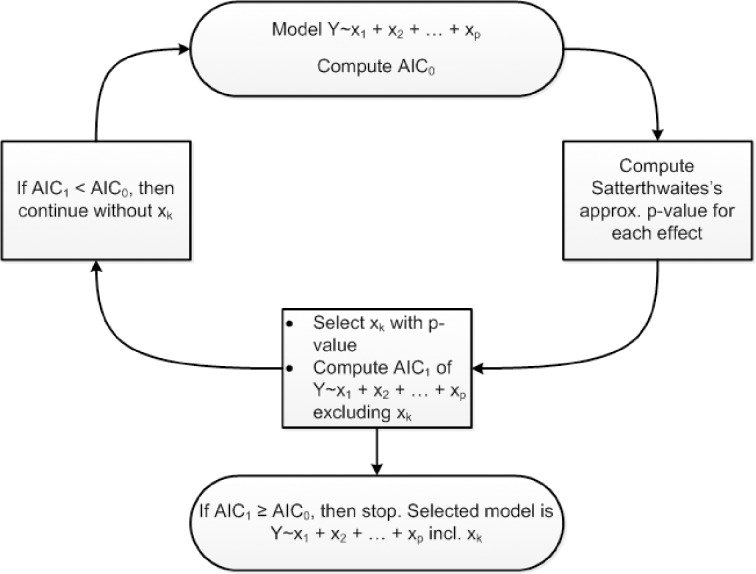
Flowchart of the model selection procedure.

We used linear mixed models for the statistical analysis of the data. The analysis of the regional volume data was performed with the volumes as response variable (*V*). The models consisted of fixed main effects and fixed interaction effects which we selected due to their suspected influence on hippocampal volume and shape. Fixed main effects were segmentation method (*M*) with levels (Manual, FSL-FIRST, FreeSurfer), Group (*G*) with levels (CTRL, MCIN, MCIP, AD), hemisphere (*H*) with levels (Left, Right), region (*R*) with levels (Anterior, Middle, Posterior) and time-point (*T*) with levels (BL, M12). A complete model would include all combinations of pairs, triples, etc. of these effects. To reduce the model complexity we started our search for a physiologically reasonable descriptive model by only considering the following interactions: group-method (*G-M*), group-region (*G-R*), method-region (*M-R*), method-hemisphere (*M-H*), time-point-group (*T-G*), time-point-region (T-R), time-point-group-region (T-G-R) and group-region-method (*G-R-M*). Individual subject effects (*S*) were modelled as random effects. This yielded the mixed model:

V∼M+G+H+R+T+GM+GR+MR+MH+TG+TR+TGR+GRM+r(S) (8)

where *r()* indicates a random effect. This model was fitted to the pair of longitudinal A scans and the pair of B scans separately. Then a model selection algorithm was run that selected significant effects amongst fixed effects present in the model. This was done by minimizing the Akaike Information Criterion (AIC) in a backward elimination set up, i.e. least significant terms were dropped from the model until the AIC started to increase. The AIC is a commonly used statistical measure that balances the goodness of fit and model complexity (i.e. number of free parameters). Significance of each term was computed according to an ANOVA analysis with Satterthwaite’s approximation for degrees of freedom using R-package lmerTest [46]. The model selection is illustrated with a flowchart in Fig 3.

For the analysis of whole hippocampus outline reproducibilities we transformed the Jaccard index in (2) to *Jacc*^*9*^ as response variable (*J*) in order to fulfil the assumption of Gaussian errors in the linear model. Fixed main effects were segmentation method (*M*), group (*G*), hemisphere (*H*), time-point (*T*). Fixed interaction effects fitted were group-method (*G-M*) and method-hemisphere (*M-H*). Individual Subject effects (*S*) were modelled as random effects. In all this yielded the mixed model:

J∼G+M+H+T+GM+MH+r(S) (9)

The regional hippocampus outline reproducibilities were analysed in a similar way. The Jaccard index in (3) was again transformed to *Jacc*^*9*^ as response variable in order to meet the Gaussian assumption. Compared to the whole hippocampus analysis we added the fixed main effect Region (*R*) and interaction effects R-M and R-G:

$$J_{ROI}∼G+M+H+T+R+GM+MH+RM+RG+r(S)$$ (10)

The model selection for (9) and (10) was performed using the same algorithm as used for the volume data analysis. For the volume data analysis FreeSurfer’s segmentations from the longitudinal stream have been used, but for comparison we also analysed segmentations from the cross-sectional stream. The reproducibility analysis was only performed with FreeSurfer’s segmentations from the cross-sectional stream.

## Results

### Registration quality control

**Fig 4:**
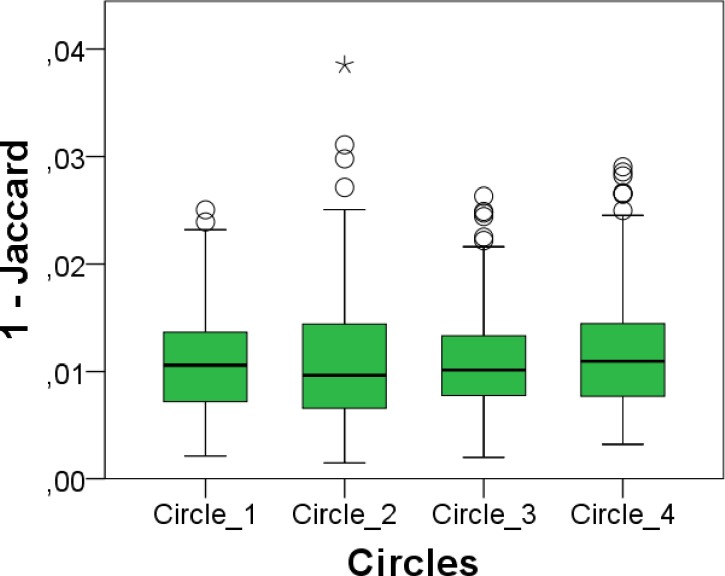
Quality control for MRI scan registration. Results obtained from the residual matrix after the full circle approach. Jaccard error computed for circles defined by Eq 7.

Quality of the registrations for all subjects was analysed using the full circle method to evaluate the transitivity error. Taking all subjects into account, for the full circles of the primary analysis, described by equations in (4) the maximum total rotation and translation calculated were 0.12deg and 0.4mm respectively. The mean translation and rotation were 0.01 mm and 0.04 degrees, which is the result of three registration steps, so that each registration will be more accurate than this. In Fig 4. the registration error is plotted in boxplots showing the error for each circle on the basis of Eq (7). In general, all values are quite small, demonstrating the consistency and accuracy of registrations. Additionally, registrations of outliers shown in Fig 4. were reviewed visually and showed no noticeable registration errors, which indicated together with rotation and translation results that all registrations were of good quality.

### Regional hippocampus volume comparison

**Fig 5:**
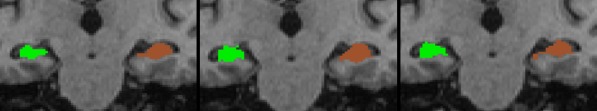
Left and right hippocampus segmentations in coronal view. Left: manual segmentation. Middle: FSL-FIRST segmentation. Right: FreeSurfer segmentation.

For the regional analysis segmented hippocampi of all segmentation methods, shown in Fig 5, have been processed as described in chapter 2.3. Regional volumes have been extracted and used for our statistical analysis.

The linear mixed models fitted on the BL-A and M12-A scans on the one hand and those fitted on the BL-B and M12-B scans on the other hand yielded identical selections of fixed effects. That means that in both cases the model selection procedure reduced the model of Eq (8) to the following:

V∼G+M+H+T+R+GR+MR+r(S) (11)

We then performed the model selection on all scans BL-A, M12-A, BL-B and M12-B together, and obtained again the same selection of fixed effects. In the sequel, parameter estimates from the combined data set will be mentioned. All fixed main effects and fixed interaction effects in (11) were significant, with the highest p-value (Satterthwaite’s approximation) in the selected model of 0.0001082 (main effect Group (*G*)), all other p-values were lower. The dropped fixed main effect and fixed interaction effect were insignificant and had a higher Satterthwaite’s approximation p-value than 0.05. For the factors hemisphere (*H*) and time-point (*T*) only the main effects are present in the final model and the interaction effects of these dropped. The left hippocampus was on average 0.0332cm^3^ smaller than the right hippocampus. Hippocampi from time-point M12 were on average 0.0326cm^3^ smaller than from time-point BL. Predictions of the estimated model for the three segmentation methods are shown in Table 1 for the left hemisphere and time-point BL.

**Table 1:** Predicted volumes (cm^3^) for the left hippocampus at time-point BL for all segmentation methods.

Group	CTRL	MCIN	MCIP	AD
Region				
	**Manual Segmentation**			
Anterior	1.317	1.171	1.088	1.066
Middle	1.282	1.252	1.120	1.006
Posterior	0.790	0.747	0.731	0.618
	**FSL-FIRST Segmentation**			
Anterior	1.259	1.113	1.031	1.008
Middle	1.369	1.340	1.207	1.093
Posterior	0.964	0.920	0.905	0.792
	**FreeSurfer Segmentation**			
Anterior	1.186	1.040	0.957	0.935
Middle	1.324	1.294	1.162	1.048
Posterior	1.000	0.957	0.942	0.828

**Fig 6:**
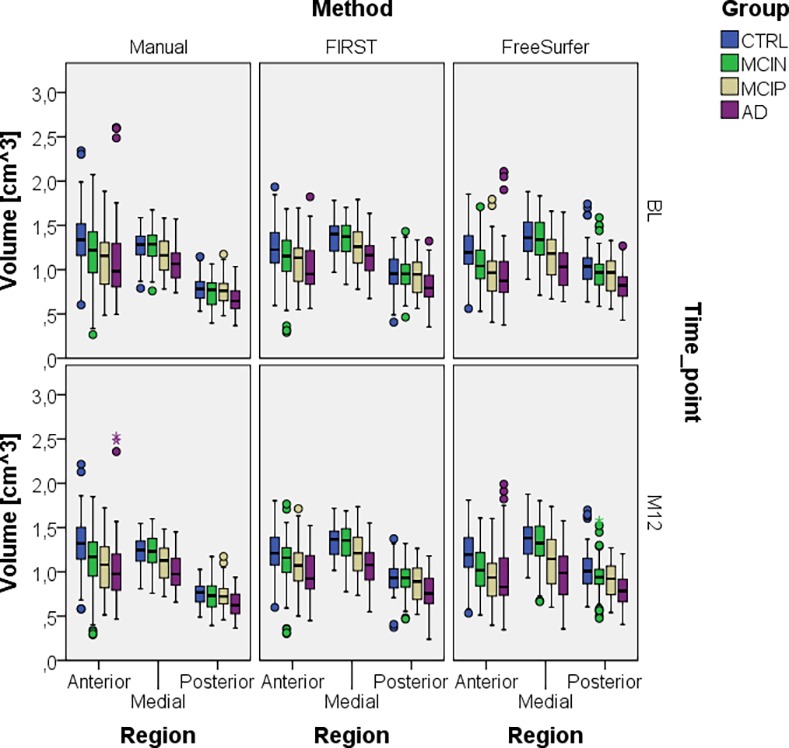
Regional volume comparison for all methods and both time-points. Left and right hippocampus and scan A and B were grouped together.

Using the average volume difference between left and right (0.0332cm^3^) or between BL and M12 (0.0326cm^3^) hippocampi, all other predicted volumes can be reconstructed by adding these values to the predicted volumes in Table 1. For example, to obtain the predicted volume from the FSL-FIRST segmentations in the MCIP group of the middle region for the right hippocampus, 0.0332cm^3^ need to be added to 1.207cm^3^. Tables for right hippocampus at time-point BL and left and right hippocampus at time-point M12 can be found in the supporting information (Table in S1 Table, Table in S2 Table and Table in S3 Table). The decrease of volume from BL to M12 could be predicted by all methods, but could not be differentiated between different group types. In general, the middle part for both automatic segmentation methods was the largest part, while for manual segmentations the anterior and middle parts seem to be of almost equal size. Moreover, the anterior volume of manual segmentations was systematically bigger than the anterior volume of the automatic segmentations. Also noticeable is that for all three methods the posterior part was predicted to be the smallest part, which is the result of our definition of the ROIs within the mask. Furthermore, the predicted volumes from Table 1 shows that for all methods all three regions showed a decrease in hippocampal volume for increasing severity of disease. Fig 6 illustrates regional hippocampal volume differences for all three methods and regions discriminated in groups and by both time-points, while left and right hippocampi were grouped together.

Following the same procedure, using FreeSurfer’s segmentation from the cross-sectional stream resulted in the same model with very similar predicted volumes, which can be found in the supporting information (Table in S4 Table).

### Whole hippocampus outline reproducibility

The fitted and selected linear mixed model for the hippocampus outline reproducibility only contains the fixed effect method (M), with p-value <2.2x10^-16^. The predicted Jaccard indices for the three segmentation methods are shown in Table 2. This table shows that FSL-FIRST segmentation is the most and FreeSurfer segmentation the least reproducible.

**Table 2:** Predicted Jaccard indices for the whole hippocampus for the different segmentation methods.

Method	Jaccard
Manual	0.795
FSL-FIRST	0.829
FreeSurfer	0.754

**Fig 7:**
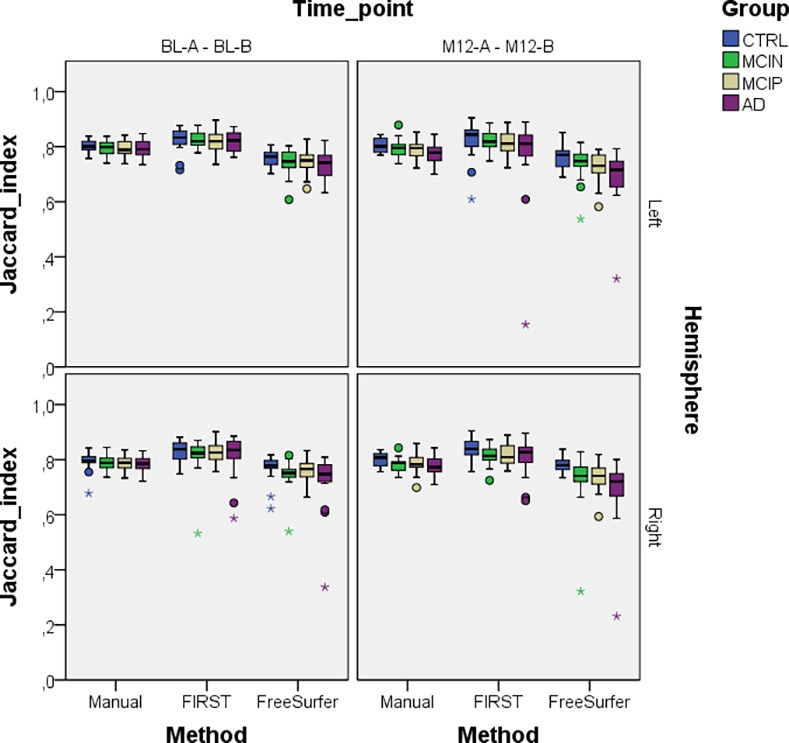
Whole hippocampus Jaccard indices for all methods, both time-points and left and right hippocampi to show segmentation reproducibility between BTB scans.

Fig 7 illustrates Jaccard indices of outline reproducibility for all three methods for BL and M12 scan pairs, separated by left and right hippocampus and differentiated into groups. The boxplots show the same tendency as predicted by the mixed model. Even though it was not significant, the boxplots also show a trend that for all methods Jaccard indices decrease with increasing disease severity, and both automatic segmentations show larger variations than manual segmentations. Also, it should be noted that only the automatic segmentations have large outliers.

### Regional hippocampus outline reproducibility

Regional hippocampus Jaccard indices have been computed by using Eq (3). The fitted linear mixed models contain as fixed effects the main effects method and region and interaction effect region-method, resulting into the model:

$$J_{ROI}∼M+R+RM+r(S)$$ (12)

The p-values of all three fixed effects in the selected model were similar to the p-values of the analysis of the whole hippocampus outline reproducibility. The predicted Jaccard indices for all method-region combinations are shown in Table 3.

**Table 3:** Predicted Jaccard indices for the regional hippocampus for the different segmentation methods.

Region	Anterior	Middle	Posterior
Method			
Manual	0.794	0.825	0.756
FSL-FIRST	0.829	0.855	0.798
FreeSurfer	0.756	0.784	0.721

**Fig 8:**
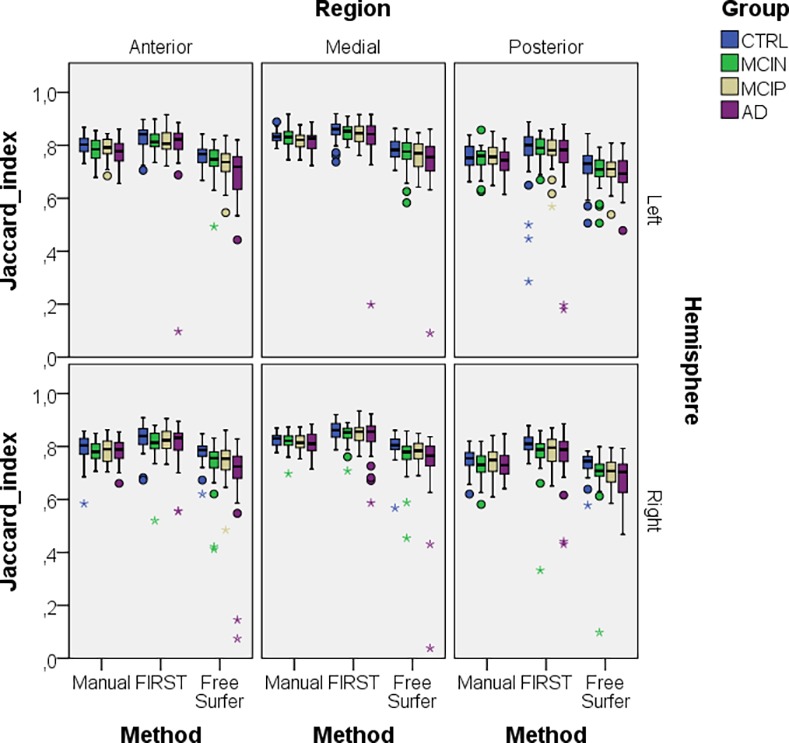
Regional hippocampus Jaccard indices for all methods and left and right hippocampi. We combined both time-points, i.e. BL-A–BL-B and M12-A– M12-B, because both time-points by themself gave similar results.

The results related to the segmentation method are similar to that in the whole hippocampus analysis: FSL-FIRST segmentation is most and FreeSurfer segmentation least reproducible. It can also be seen in Fig 8 that for all methods with the severity of the disease in all regions the reproducibility decreased. Additionally, Table 3 shows that the middle region has highest Jaccard indices and the posterior region lowest Jaccard indices.

## Discussion and conclusion

With our approach to automatically and precisely extract regional hippocampal volumes and outline reproducibilities from the BTB scans’ segmentations we were able to detect systematic differences in volumes among three different segmentation methods and showed that FSL-FIRST was the most reproducible segmentation method.

In several applications, the quantification of global hippocampal volumes is of limited applicability. For instance, when studying anatomical changes accompanying the development of neurodegenerative diseases or when testing drugs against these diseases, it is well possible that these changes occur in specific regions of the hippocampus and then global measures such as volume would be too coarse to notice them. For clinical applications in radiotherapy where hippocampus avoidance is aimed for, it is insufficient to know that volume of the delineated object is correct, but also accuracy of shape is required. Finally, the need for local shape information is required to determine whether differences in hippocampus segmentation by different methods are caused by hidden systematic differences in the underlying anatomical definitions of the hippocampus.

The present study developed a method to investigate regional effects in shape differences. Confirming with other literature [26,47–51], also our analysis showed a global left and right hippocampus difference. Furthermore, global hippocampal atrophy could be detected, but it could not be distinguished in between groups (G) or regions (R), because the interaction of these with the time-point (T) were not significant. The regional volume analysis showed that both automatic segmentations revealed similar results, while manual segmentations had systematically larger anterior, and smaller middle and posterior volume predictions, which indicates that the hippocampus segmentation protocol for manual segmentations is different than the definition of the hippocampus underlying the automatic segmentation methods. Both, FSL-FIRST and FreeSurfer subcortical segmentations are based on manually labelled training data sets following the outline protocol from the Center of Morphometric Analysis (CMA, http://www.cma.mgh.harvard.edu/). The intention of both the hippocampal outlining protocol of the CMA and that of Jack and colleagues [41] used in this study for manual segmentation, is to include: dentate gyrus, cornu ammonis, subiculum, fimbria and alveus. Alterations of regional volume distributions among methods shows that with our analysis more subtle differences in segmentation protocols were detectable. Therefore, it would be beneficial to use a standardized protocol like the harmonized protocol for hippocampus volumetry, the outcome of a project to define a standard protocol for hippocampus segmentation [52][53][54].

With our regional volume data we also compared FreeSurfer’s results from the cross-sectional and longitudinal stream. For both we obtained the same model with the same selection of fixed effects, only the predicted volumes differed: FreeSurfer’s anterior and posterior volume predictions were slightly larger for results from the longitudinal stream. Even though Reuter and colleagues [15] showed an improvement in distinguishing diagnostic groups using the longitudinal stream, with our approach the selected model using either the cross-sectional or longitudinal stream was identical, i.e. neither increased reproducibility nor accelerated decrease of hippocampal volume in AD subjects were found when using the a priori knowledge that scans form a longitudinal series. This might be due to the smaller number of subjects used in this study, as Reuter and colleagues used three times as many non-demented and demented subjects.

At the IAC Amsterdam, technicians undergo yearly reliability trainings with training sets of five cases. In the most recent test sets, the intra-rater variability score of the hippocampal volume—ICC with absolute agreement—was 0.985–0.99 using identical images. Determining the ICC with absolute agreement measure using the BTB dataset of the current study, the technician obtained an ICC of 0.98 and 0.99 for hippocampal volumes of BL-A–BL-B and M12-A– M12-B scans respectively. For FSL-FIRST the ICC was 0.98 and 0.98 and for FreeSurfer it was 0.99 and 0.98 for BL and M12 BTB scans respectively. Even though hippocampal volumes have high correlations, our outline reproducibility analysis showed that comparing volumes alone does not reflect the complete picture of the quality of the outline. We determined outline reproduciblities for the whole hippocampus, but also for anterior, middle and posterior hippocampus sections. For both left and right hippocampus, whole hippocampus and in all three subregions, in all diagnostic groups and at both time points, FSL-FIRST consistently gave significant higher Jaccard indices, followed by manual, followed by FreeSurfer. This confirms the finding of Morey and colleagues, who also found that FSL-FIRST had higher outline reproducibilities than FreeSurfer [30]. However, it should be mentioned that only automatic segmentation methods had large outlier Jaccard indices, as can be seen in Figs 7 and 8. To confirm that these truly resulted from poor segmentations and not by registration errors we visually inspected the MRI scan pairs of these outliers as described in 2.4. No visual noticeable registration errors could be detected, but poor segmentations could be confirmed by inspecting the mesh segmentations of these outliers. The hippocampal volumes of these outliers were also reviewed but they did not show outlier values.

With our regional reproducibility analysis we were also able to determine that for all segmentation methods the middle region had highest Jaccard indices. The middle region shares common borders with the anterior and posterior region, which means the border surface of the middle region to other structures is smaller compared to the anterior and posterior regions. Due to similar grey values the hippocampus is hard to distinguish from adjacent structures, which means the regions with a larger surface to adjacent structures most probably have a poorer reproducibility, as it can be seen from the anterior and posterior region. It should also be noted that overlap indices in general are sensitive to size differences. The size differences between anterior and posterior parts amounted to between 15 to 20% (Table 1), which could therefore also provide a partial explanation for the observed differences in Jaccard indices.

Given that reproducibility is an important requirement for segmentation methods, FSL-FIRST meets the requirement and exhibits even better results than manual outlining, which is the choice of many clinical trials. Nevertheless, this finding should be treated with care, because outline reproducibility is necessary, but not sufficient to imply that the hippocampus was outlined accurately. In contrast, E. Mulder and colleagues [33] found that FreeSurfer obtains most reproducible volume atrophy measurements compared to manual and FSL-FIRST segmentations. Considering that we found that FreeSurfer has worst outline reproducibilities atrophy measurements FreeSurfers’ hippocampus segmentations should be interpreted with care. Furthermore, results show that for all methods and subregions, and for both hemispheres and both time points, AD patients tend to exhibit poorer reproducibilities than healthy controls, while especially FreeSurfers’ results have larger decrease in Jaccard indices with disease severity than manual and FIRST segmentations; and only automatic segmentation methods showed extreme Jaccard indices. This finding was not detected as a significant effect by our statistical model because the variation was too large for our sample size. But it is an indication that the training sets of the automatic methods might not be optimized for diseased subjects, which is confirmed by several other studies [20][22][31].

In this study we also proposed a novel method to extract regional Jaccard indices by converting label images to meshes and by using registration parameters on these meshes to map them to a common space. This approach is particularly useful when comparing small structures, because interpolation and registration errors are avoided. The full circle method allowed us to quantitatively estimate registration accuracy by computing rotation and translation components, but we also extended this method to a consistency measure using the Jaccard index. We suggest that this methodology can be a useful tool in other (brain) imaging studies where small structures are compared between scans with different image orientations.

For a better disease understanding and more sophisticated analysis it would be an idea to extend the regional analysis to more specific hippocampal subfields (cornu ammonis fields, dentate gyrus and subiculum). This is an ongoing field of interest and usually high field scanners over 3T with high resolution T2 or proton density sequences are necessary to distinguish boundaries between those regions [55]. We suggest that for the analysis of such datasets the methodology proposed in this study would be particularly suited.

## Supporting information

S1 Table Predicted volumes (cm^3^) for the right hippocampus at time-point BL for all segmentation methods. (DOCX)

S2 Table Predicted volumes (cm^3^) for the left hippocampus at time-point M12 for all segmentation methods. (DOCX)

S3 Table Predicted volumes (cm^3^) for the right hippocampus at time-point M12 for all segmentation methods. (DOCX)

S4 Table Volume predictions (cm3) for the left hippocampus at time-point BL using FreeSurfer’s segmentations for the cross-sectional stream. (DOCX)
