# Supplementary material for: Regional analysis of volumes and reproducibilities of automatic and manual hippocampal segmentations
Source: PLoS One. 2017 Feb 9;12(2):e0166785. doi: 10.1371/journal.pone.0166785 (PMC5300281; doi:10.1371/journal.pone.0166785)
Supplement: S1 Table — (DOCX) [file pone.0166785.s001.docx]

S1 Table: Predicted volumes (cm^3^) for the right hippocampus at time-point BL for all segmentation methods.

| **Region**  **Group** | CTRL | MCIN | MCIP | AD |
| --- | --- | --- | --- | --- |
|  | **Manual Segmentation** | | | |
| Anterior | 1.350 | 1.204 | 1.121 | 1.099 |
| Middle | 1.315 | 1.285 | 1.153 | 1.039 |
| Posterior | 0.823 | 0.780 | 0.764 | 0.651 |
|  | **FSL-FIRST Segmentation** | | | |
| Anterior | 1.292 | 1.146 | 1.064 | 1.041 |
| Middle | 1.402 | 1.373 | 1.240 | 1.126 |
| Posterior | 0.997 | 0.953 | 0.938 | 0.825 |
|  | **FreeSurfer Segmentation** | | | |
| Anterior | 1.219 | 1.073 | 0.990 | 0.968 |
| Middle | 1.357 | 1.327 | 1.195 | 1.081 |
| Posterior | 1.033 | 0.990 | 0.975 | 0.861 |
