# Supplementary material for: Regional analysis of volumes and reproducibilities of automatic and manual hippocampal segmentations
Source: PLoS One. 2017 Feb 9;12(2):e0166785. doi: 10.1371/journal.pone.0166785 (PMC5300281; doi:10.1371/journal.pone.0166785)
Supplement: S2 Table — (DOCX) [file pone.0166785.s002.docx]

S2 Table: Predicted volumes (cm^3^) for the left hippocampus at time-point M12 for all segmentation methods.

| **Region**  **Group** | CTRL | MCIN | MCIP | AD |
| --- | --- | --- | --- | --- |
|  | **Manual Segmentation** | | | |
| Anterior | 1.284 | 1.138 | 1.055 | 1.033 |
| Middle | 1.249 | 1.219 | 1.087 | 0.973 |
| Posterior | 0.757 | 0.714 | 0.698 | 0.585 |
|  | **FSL-FIRST Segmentation** | | | |
| Anterior | 1.226 | 1.080 | 0.998 | 0.975 |
| Middle | 1.336 | 1.307 | 1.174 | 1.060 |
| Posterior | 0.931 | 0.887 | 0.872 | 0.759 |
|  | **FreeSurfer Segmentation** | | | |
| Anterior | 1.153 | 1.007 | 0.924 | 0.902 |
| Middle | 1.291 | 1.261 | 1.129 | 1.015 |
| Posterior | 0.967 | 0.924 | 0.909 | 0.795 |
