# Supplementary material for: Regional analysis of volumes and reproducibilities of automatic and manual hippocampal segmentations
Source: PLoS One. 2017 Feb 9;12(2):e0166785. doi: 10.1371/journal.pone.0166785 (PMC5300281; doi:10.1371/journal.pone.0166785)
Supplement: S3 Table — (DOCX) [file pone.0166785.s003.docx]

S3 Table: Predicted volumes (cm^3^) for the right hippocampus at time-point M12 for all segmentation methods.

| **Region**  **Group** | CTRL | MCIN | MCIP | AD |
| --- | --- | --- | --- | --- |
|  | **Manual Segmentation** | | | |
| Anterior | 1.317 | 1.171 | 1.088 | 1.066 |
| Middle | 1.282 | 1.252 | 1.120 | 1.006 |
| Posterior | 0.790 | 0.747 | 0.731 | 0.618 |
|  | **FSL-FIRST Segmentation** | | | |
| Anterior | 1.259 | 1.113 | 1.031 | 1.008 |
| Middle | 1.369 | 1.340 | 1.207 | 1.093 |
| Posterior | 0.964 | 0.920 | 0.905 | 0.792 |
|  | **FreeSurfer Segmentation** | | | |
| Anterior | 1.186 | 1.040 | 0.957 | 0.935 |
| Middle | 1.324 | 1.294 | 1.162 | 1.048 |
| Posterior | 1.000 | 0.957 | 0.942 | 0.828 |
