# Supplementary material for: Regional analysis of volumes and reproducibilities of automatic and manual hippocampal segmentations
Source: PLoS One. 2017 Feb 9;12(2):e0166785. doi: 10.1371/journal.pone.0166785 (PMC5300281; doi:10.1371/journal.pone.0166785)
Supplement: S4 Table — (DOCX) [file pone.0166785.s004.docx]

S4 Table: Volume predictions (cm3) for the left hippocampus at time-point BL using FreeSurfer’s segmentations for the cross-sectional stream. Using the average volume difference between left and right or between BL and M12 hippocampi (both 0.034cm3), all other predicted volumes can be reconstructed by adding these values to the predicted volumes in S4Table.

| **Region**  **Group** | CTRL | MCIN | MCIP | AD |
| --- | --- | --- | --- | --- |
|  | **Manual Segmentation** | | | |
| Anterior | 1.315 | 1.169 | 1.090 | 1.067 |
| Middle | 1.283 | 1.250 | 1.122 | 1.005 |
| Posterior | 0.791 | 0.745 | 0.729 | 0.622 |
|  | **FSL-FIRST Segmentation** | | | |
| Anterior | 1.257 | 1.112 | 1.032 | 1.009 |
| Middle | 1.370 | 1.337 | 1.210 | 1.093 |
| Posterior | 0.964 | 0.918 | 0.903 | 0.795 |
|  | **FreeSurfer Segmentation** | | | |
| Anterior | 1.167 | 1.022 | 0.942 | 0.919 |
| Middle | 1.328 | 1.295 | 1.167 | 1.050 |
| Posterior | 0.965 | 0.919 | 0.903 | 0.796 |
